# Blackening of the Teeth by Antipyrine

**Published:** 1892-06

**Authors:** 


### Blackening of the Teeth by Antipyrine.

It is asserted that the internal use of antipyrine blackens the teeth; this peculiarity should be generally known by the profession, and also among the laity, that objections may be made on this ground to taking it as a remedy. The blackening is the more intense, the more imperfect the enamel, but may be removed by attrition with dilute acid. The considerable use of antipyrine for several years back, gives importance to this latter observation.—*Southern Dental Journal.*

